# Evolutionary Conservation of *pou5f3* Genomic Organization and Its Dynamic Distribution during Embryogenesis and in Adult Gonads in Japanese Flounder *Paralichthys olivaceus*

**DOI:** 10.3390/ijms18010231

**Published:** 2017-01-23

**Authors:** Jinning Gao, Xubo Wang, Quanqi Zhang

**Affiliations:** 1 College of Marine Life Science, Ocean University of China, Key Laboratory of Marine Genetics and Breeding, Ministry of Education, Qingdao 266003, China; gjn.1127@163.com; 2 Center for Developmental Cardiology, Institute for Translational Medicine, College of Medicine, Qingdao University, Qingdao 266021, China

**Keywords:** Japanese flounder (*Paralichthys olivaceus*), *pou5f3*, promoter analysis, expression pattern

## Abstract

Octamer-binding transcription factor 4 (Oct4) is a member of POU (Pit-Oct-Unc) transcription factor family Class V that plays a crucial role in maintaining the pluripotency and self-renewal of stem cells. Though it has been deeply investigated in mammals, its lower vertebrate homologue, especially in the marine fish, is poorly studied. In this study, we isolated the full-length sequence of *Paralichthys olivaceus pou5f3* (*Popou5f3*), and we found that it is homologous to mammalian *Oct4*. We identified two transcript variants with different lengths of 3′-untranslated regions (UTRs) generated by alternative polyadenylation (APA). Quantitative real-time RT-PCR (qRT-PCR), in situ hybridization (ISH) and immunohistochemistry (IHC) were implemented to characterize the spatial and temporal expression pattern of *Popou5f3* during early development and in adult tissues. Our results show that *Popou5f3* is maternally inherited, abundantly expressed at the blastula and early gastrula stages, then greatly diminishes at the end of gastrulation. It is hardly detectable from the heart-beating stage onward. We found that *Popou5f3* expression is restricted to the adult gonads, and continuously expresses during oogenesis while its dynamics are downregulated during spermatogenesis. Additionally, numerous *cis*-regulatory elements (CRE) on both sides of the flanking regions show potential roles in regulating the expression of *Popou5f3*. Taken together, these findings could further our understanding of the functions and evolution of *pou5f3* in lower vertebrates, and also provides fundamental information for stem cell tracing and genetic manipulation in *Paralichthys olivaceus*.

## 1. Introduction

Octamer-binding transcription factor 4 (Oct4) is a member of the POU transcription factor family Class V, which is essential for maintaining the pluripotency and self-renewal of stem cells. The basic feature of Oct4 is a highly conserved POU domain with two structurally independent subdomains, POUs and POUh [1]. As with other Oct proteins, Oct4 can recognize and bind to the octamer motif through its DNA-binding segments on the POU domain to activate or inhibit the transcription of downstream target genes [2]. It can also cooperatively interact with Sox2 to form complexes by binding to Oct-Sox motif to drive pluripotent-specific gene expression [3,4]. In fact, pluripotent stem cell identity is governed by the combinatorial control of transcriptional modulators Oct4, Sox2 and Nanog, and imbalance of any of them causes cells to enter a specific differentiation program [5,6,7,8].

In mice, *Oct4*/*Pou5f1* is highly expressed in pluripotent cells and germ cells of the early embryo. The expression of *Oct4*/*Pou5f1* is activated at the four-cell to morula stage and maintained in the inner cell mass (ICM) of the blastocyst while rapidly downregulated in the trophectoderm. After implantation, *Oct4*/*Pou5f1* is differentially expressed in the epiblast, decreased during gastrulation and exclusively confined to the primordial germ cells (PGCs) [9,10,11]. Although *Oct4*-deficient mouse embryos are able to develop to the blastocyst stage, their ICMs are not pluripotent and differentiate into trophectoderm [12]. A specific expression of *Oct4* among mouse adult tissues is observed in the germ stem cells [13,14]. In contrast to the ICM localization of *Oct4* in mammals, the homologue of *Oct4* in lower teleosts, which was recently renamed *pou5f3*, is spatially expressed throughout the blastomere from fertilized egg to gastrulae [15,16,17,18,19]. In addition to pluripotency regulation, zebrafish *pou5f3* (originally named pou2/spg) also has some new roles as well as partial function loss when compared with other species. It has a different expression pattern after the gastrula stage, which is expressed in developing brain and plays an important role in regionalization [16]. Moreover, the expression of *pou5f3* has not been detected in PGCs, suggesting that it is not a necessary factor for PGC development in zebrafish [20]. Though minor differences exist between zebrafish and medaka model fish systems, medaka *pou5f3* (previously called *oct4*/*pou2*) shows a similar expression pattern with mammalian *Oct4* [17,21]. A study found that the expression of *pou5f3* in differentiated medaka embryonic stem cells (ESCs) is reduced more than four-fold compared to their level in undifferentiated state ESCs, which reveals its crucial role in maintaining stem cell pluripotency [22]. Moreover, medaka *pou5f3* is able to reprogram somatic cells into pluripotent cells [23].

*Paralichthys olivaceus*, popularly known as Japanese flounder, is a mariculture flatfish species with ecological and economical importance in China. Over years of improved variety selection and breeding, new demands for the protection of genetic resources have been made. Fish embryonic stem (ES) cell lines have been demonstrated to have many common features compared to mammalian ESCs, that can be serially passaged to a stable cell line and differentiate into various types of cells, including germ cells [24]. Thus, pluripotent stem cells are considered to be the best medium for the preservation of improved varieties due to their good biological characteristics. Thus far, several ES (or ES-like) cell lines have been successfully established by using the blastula stage embryos in medaka, gilthead sea bream, sea bass, perch, India carp and Pacific cod [25,26,27,28,29,30]. However, there are as of yet no reports on Japanese flounder stem cells and their manipulation lacks theoretical reference. Therefore, the analysis of stem cell regulators that are homologous to mammals in flounder will be helpful. In this study, we identified and characterized the sequence features and expression patterns of *pou5f3* to lay a foundation for further investigation in *P. olivaceus*.

## 2. Results

### 2.1. Molecular Characterization and Homological Analysis of P. olivaceus pou5f3

**Figure 1:**
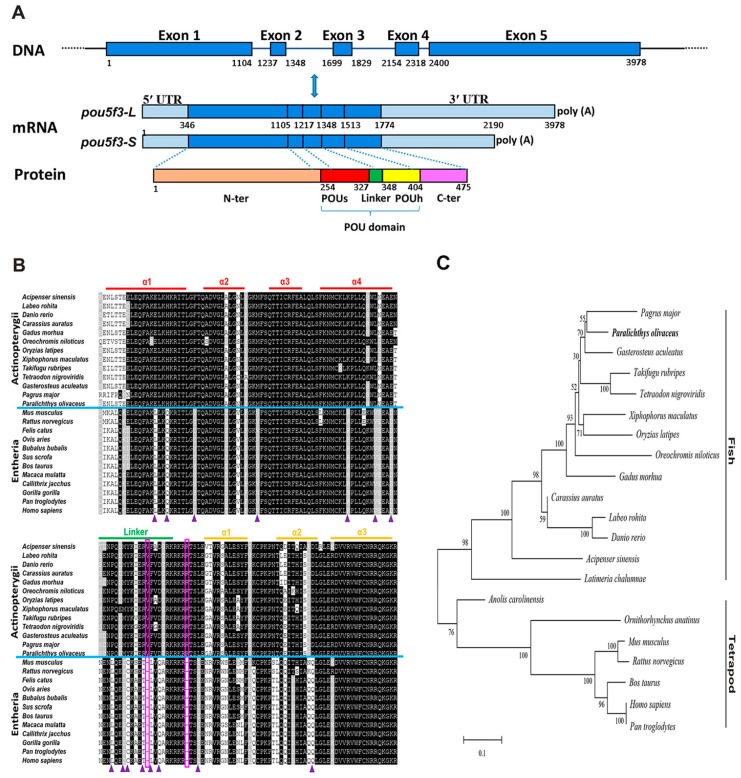
Sequence analysis of *pou5f3* gene in Japanese flounder. (**A**) The *pou5f3* gene is composed of five exons and four introns. Two isoforms of the *pou5f3* mRNA transcript (*pou5f3*-*L* and *pou5f3*-*S*) have different lengths of 3′-UTR. The schematic diagram was drawn to scale and the relative sequence numbers were indicated. UTRs (light blue); exons (dark blue). N-ter, N-terminal domain (orange); POUs, POU specific domain (red); Linker, Linker domain (green); POUh, POU homeodomain (yellow); C-ter, C-terminal domain (purple); (**B**) Amino acid sequence alignment of the POU domains from POUV proteins in various vertebrates. Identical residues and conservative substitutions are indicated in black and gray, respectively. The upper lines indicate the amino acid sequences stretched for α-helix of the POUs and POUh domains, as well as the linker region. The solid triangles and the two rectangles indicate residues mentioned in subsection 3.1; and (**C**) Phylogenetic analysis of *Po*Pou5f3 and other POUV proteins. The tree was constructed by MEGA 6.0 using Poisson correction distance based upon the neighbor-joining (NJ) method. The *Paralichthys olivaceus* Pou5f3 is highlighted in bold. The numbers at the nodes corresponding to the bootstrap support are expressed as the percentage of 1000 replicates. The GenBank accession numbers or Ensembl IDs of analyzed sequences are listed in Table S1.

We have cloned *P. olivaceus pou5f3* from the blastula-stage embryos through the homology cloning method and the rapid-amplification of cDNA ends (RACE) strategy. It exhibited two transcript isoforms with an identical 5′-UTR (345 bp length) and different lengths of 3′-UTR, which are 874 and 1318 bp long, respectively. The deduced *Po*Pou5f3 contained an open reading frame (ORF) with 1428 bp encoding a protein of 475 amino acids with a calculated molecular mass (MM) of 51.98 kDa and a theoretical isoelectric point (PI) of 6.28 (GenBank accession number: KJ522774) (Figure 1A and Figure S1). Similar to other previously identified Class V POU proteins, the predicted *Po*Pou5f3 possessed a characteristic POU domain consisting of the POU specific domain, the POU homeodomain and a linker region between them (Figure 1 and Figure S2).

The analysis for sequence homology showed that *Po*Pou5f3 has 37.2% to 81.5% amino acid sequence identities and the conserved POU domain of *Po*Pou5f3 showed 68.5% to 97.4% identities to those from other vertebrates (Table 1). The alignment of *Po*Pou5f3 with other POUV proteins from mammals, avian, reptile, amphibian, and teleosts showed that conservation was high at the POU domain when compared to the full-length protein. Moreover, *Po*Pou5f3 shares a significantly higher sequence identity with other teleosts than tetrapods (Table 1). In addition, there were two one-residue deletions located within the linker region and the POU homeodomain, and several distinct residues in the POU domain when compared with actinopterygii POU5F3 to that of entheria POU5F1 (Figure 1B).

To evaluate the evolutionary relationship between the predicted *Po*Pou5f3 and other vertebrate orthologues, a phylogenetic tree that contained two main branches, fish Pou5f3 and tetrapod Pou5f1, was constructed with the full-length amino acid sequences using the neighbor-joining method (Figure 1C). Based on our identification, the *Po*Pou5f3 is obviously clustered with the fish Pou5f3 homologues and the relationships displayed in the phylogenetic tree is generally in accordance with classical taxonomy.

### 2.2. Genomic Organization and Conserved Synteny of Class V POU Family Members

**Figure 2:**
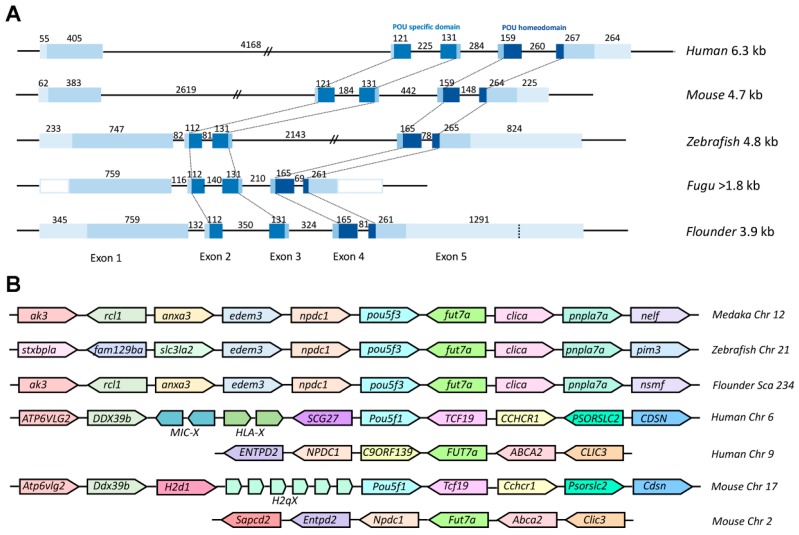
Gene structure and chromosome synteny of *Pou5f1*/*pou5f3* in vertebrate. (**A**) Comparison of genomic organizations of *Pou5f1*/*pou5f3* between fish and mammals. Exons are shown in blue box while introns are shown in straight line. The sizes of primary transcripts and each part are indicated by base pair. The 5′- and 3′-UTRs are not known for Fugu. The conserved POUs and POUh domains are connected with dot line among species; and (**B**) Schematic diagram for the loci of *Pou5f1*/*pou5f3* genes in genomes of fish, human, and mouse from ensemble database. Chr, chromosome; Sca, scaffold.

To further explore the evolutionary conservation of Class V POU genes, we performed comparative analysis between *Popou5f3* cDNA and its genomic DNA sequence. Our results revealed that it consists of five exons and four introns (Figure 1). All exon-intron boundaries conform to the GT-AG rule (Figure S1). As shown in Figure 2A, *Popou5f3* exhibited a similar genomic organization as other teleost and mammal orthologues. Importantly, the POUs and POUh domains are conservatively located within the second to third and fourth to fifth exons in all species despite their different gene sizes. Although the first exons in human and mouse are shorter than those in fugu and flounder, the first intron greatly extended them to have much longer genomic lengths. Consistent with our previously studied *nanog* gene [31], *pou5f3* in zebrafish also showed similar genomic length with mammals rather than other fish due to expanded introns. *Popou5f3* was found on scaffold 234 flanked by *npdc1* and *fut7a* according to our up-to-date *P. olivaceus* whole-genome sequencing data. A cross-species comparison of the chromosomal syntenic relationship showed that *Popou5f3*-containing region is mostly syntenic to the *pou5f3*-bearing region of other fish genomes (*edem3*-*npdc1*-*pou5f3*-*fut7a*-*clica*) (Figure 2B). However, the human and mouse *Pou5f1* were located on the other chromosome between *Ddx39b* and *Tcf19* despite their counterpart of *Popou5f3* neighbors (*Npdc1* and *Fut7a*) exhibiting a conserved synteny on human chromosome 9 and mouse chromosome 2.

### 2.3. Bioinformatics Analysis of Popou5f3 5′ and 3′ Regulatory Sequences

**Figure 3:**
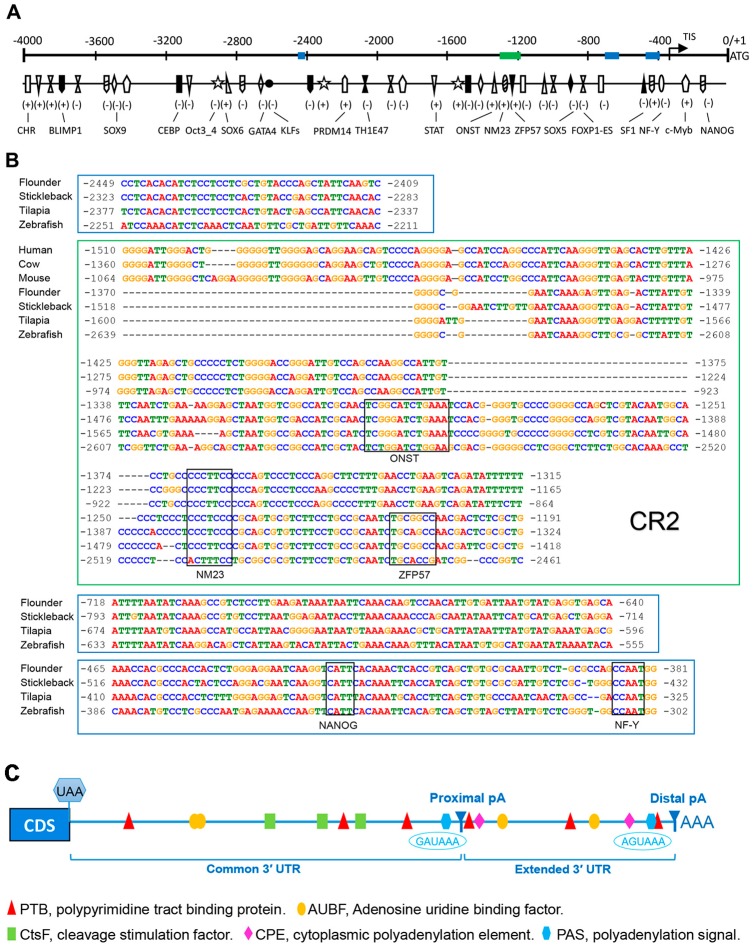
The 5′ and 3′ flanking sequences features of *Popou5f3* gene. (**A**) Sketch map of potential transcription factor (TF) binding sites in the upstream region. The map is drawn to scale with the start codon ATG designated as +1. The plus-minus sign indicates the TF binding strand. The blue and green panes represent the conserved regions among species corresponding to the sequences shown in (**B**). TIS: transcriptional initiation site; (**B**) The 5′ flanking consensus regions of *Pou5f1*/*pou5f3* orthologues among different species with the MEME and Dialign softwares. The genomic sequences were acquired from the Ensembl database and the transcript IDs are listed in Table S2. The binding sites for TFs common for all species (ONST, NM23, ZFP57, NANOG, and NF-Y) are annotated with black boxes above the aligned sequences; and (**C**) Sketch map of potential binding sites in the downstream region. The cleavage sites are shown as proximal pA and distal pA, respectively. Two non-canonical polyadenylation signals (blue hexagons) are positioned at 816 and 1265 bp downstream of the coding region. Binding sites for PTB (UCUUU), AUBF (AUUUA), CtsF (UUUUU), and CPE (UUUUAU) are represented relative to their locations with a red triangle, yellow oval, green rectangle, and pink rhombus, respectively.

To identify the potential *cis*-regulatory elements (CREs) regulating *Popou5f3* expression, we analyzed about 4000 bp genomic sequences upstream of the ATG codon using several online bioinformatics software. Consistent with the previously identified TATA-less promoter in mouse *Pou5f1* [32], we also could not find the typical TATA-box or CAAT-box in the *Popou5f3* promoter region. Nevertheless, sequence analysis with the MatInspector program revealed that there are numerous transcription factor binding sites on the regulatory region of *Popou5f3*, including POU domain class 5 transcription factor1 (Oct 3_4), homeobox protein Nanog (NANOG), PR domain zinc finger protein 14 (PRDM14), transcriptional repressor B lymphocyte-induced maturation protein 1 (BLIMP1), Kruppel-like factors (KLFs), signal transducer and activator of transcription (STAT), cellular and viral myb-like transcriptional regulator (c-Myb), Thing1/E47 heterodimer (ThTH1/E47), alternative splicing variant of forkhead box protein P1 (FOXP1-ES), and composed binding site for Oct4, Sox2, Nanog, Tcf3 and Sall4b in pluripotent cells (ONST) (Figure 3A). Additionally, binding sites for CCAAT/enhancer binding protein (C/EBP), GATA binding protein (GATA4), nuclear factor Y (NF-Y), steroidogenic factor 1 (SF1) and zinc-finger protein 57 (ZFP57) were also identified (Figure 3A). Four conserved regions were predicted among the teleost *pou5f3* promoters, but only one of them shared some homology with that of mammal *Pou5f1*, which is to be the conserved region 2 (CR2) in the mammal *Pou5f1* promoter (Figure 3B).

We obtained two transcript isoforms with different 3′-UTRs, which are generated by alternative polyadenylation (APA). In consequence, *Popou5f3* contained two poly (A) sites in the 3′ exon, distinguished as proximal- and distal-pA on the basis of their positions relative to the coding region. Meanwhile, we found that *Popou5f3* 3′-UTR has a common region and an extended region. Sequence analysis revealed that *Popou5f3* transcripts lacs the canonical polyadenylation signal (AATAAA). However, putative non-canonical polyadenylation signals are predicted upstream of the proximal and distal poly (A) sites, which were GAUAAA and AGUAAA, respectively. Other *cis*-acting regulatory elements were also identified within the common and extended 3′-UTRs, such as binding sites for polypyrimidine tract binding protein (PTB), adenosine uridine binding factor (AUBF), cleavage stimulation factor (CtsF), and cytoplasmic polyadenylation element (CPE) (Figure 3C).

### 2.4. Spatiotemporal Expression of Popou5f3 during Early Embryogensis

**Figure 4:**
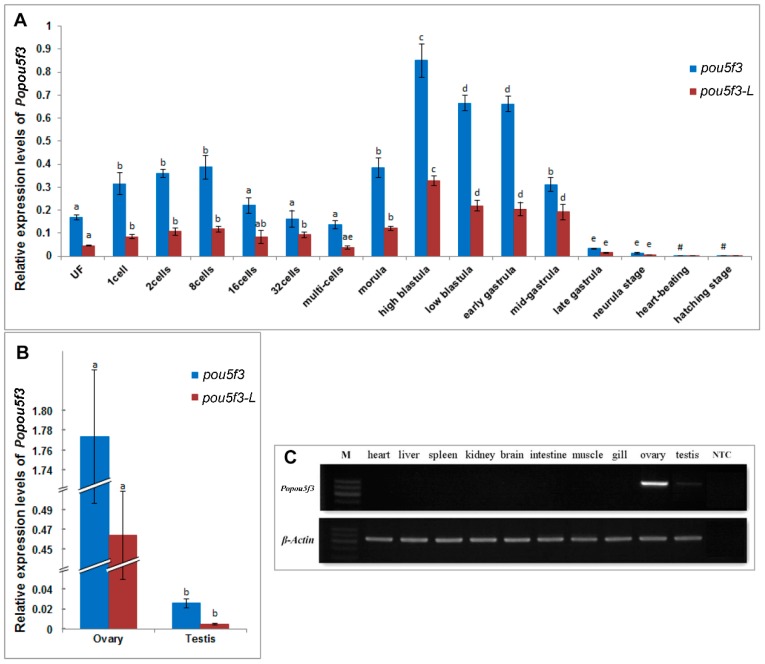
Quantitative analyses of the expression profiles of *Popou5f3*. (**A**) Relative expression levels of *Popou5f3* and the long transcript *Popou5f3-L* during early embryo development; (**B**) Relative expression levels of *Popou5f3* and *Popou5f3-L* in adult gonads. *18S RNA* and *UbcE* are chosen as the reference genes. Data are represented as mean ± SEM (*n* = 3). Groups marked with different letters are statistically different (*p* < 0.05); and (**C**) Tissue distribution of *Popou5f3* analyzed by semi-quantitative RT-PCR using *β-actin* as a reference gene. NTC, negative control without template.

Next, we investigated the expression pattern of *Popou5f3* during early embryonic development. Real-time RT-PCR analysis showed that *Popou5f3* is to be maternally expressed. The transcripts of *Popou5f3* were present from the fertilized egg to the gastrula stage of embryo (Figure 4A). With the cleavage proceeding, the amount of transcripts were increased from the one-cell to eight-cell stage and decreased from the 16-cell stage to multi-cells because of decay of maternally deposited transcripts. Its level was up-regulated again from the morula stage on, which is probably attributed to the activation of zygotic gene expression. We observed that *Popou5f3* reaches its highest expression level at the high-blastula stage; although its level decreases, it remains relatively high until the mid-gastrula stage, and then greatly diminishes at the end of the gastrulation stage. From the heart-beating stage onward, *Popou5f3* is barely detectable. Next, we analyzed the expression profile of the long transcript of *Popou5f3*, which we named *Popou5f3-L*, with specific-primers located within the extended 3’-UTR. The level of the short transcript *Popou5f3*-*S* was estimated by subtraction of the total amounts of *Popou5f3* to *Popou5f3*-*L*. Overall, *Popou5f3-L* exhibited a similar expression pattern with the total *Popou5f3* transcripts and *Popou5f3-S* showed a higher expression level than *Popou5f3-L* (Figure 4A).

**Figure 5:**
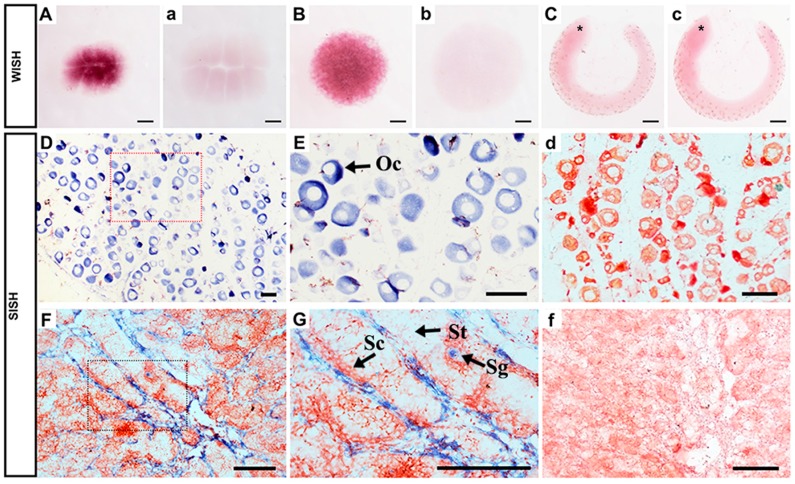
Expression of *Popou5f3* during early embryogenesis and in adult gonads analyzed by in situ hybridization (ISH). The positive signals with antisense probe hybridization were stained with dark purple or blue, while the negative controls with sense probe (**a–c**,**d**,**f**) were not stained. (**A**) eight cells, (**B**) blastula stage, (**C**) tail-bud stage. *Popou5f3* mRNA transcripts were located in the cytoplasm of oocytes in ovary (**D**,**E**). In testis, *Popou5f3* expressed in spermatogonia, but no signals in spermatocytes or spermatids (**F**,**G**). (**E**,**G**) are magnification of (**D**,**F**). Asterisks (*) indicate the head of the embryo. Oc, oocytes; Sg, Sc, and St are represent spermatogonia, spermatocytes, and spermatids, respectively. Scale bars, 100 μm.

To further investigate *Popou5f3* expression in more detail, whole-mount in situ hybridization (WISH) was performed using digoxigenin (DIG)-labeled antisense and sense probes on three selected early developmental stage embryos. A strong signal was detected at the eight-cell stage (Figure 5A), which suggests its maternal expression. At the blastula stage, the signal was present in all blastomeres (Figure 5B). At the tail-bud stage, a period between the neural and heart-beating stages, the signal was not detectable in the embryo, which is consistent with the qRT-PCR data (Figure 5C). Together, this data confirm the results of qRT-PCR. For all studied stages, no signal was observed in embryos hybridized with the sense probe (Figure 5a–c).

### 2.5. Gonad-Specific Expression of Popou5f3 in Adult Tissues

To determine the distribution of *Popou5f3* in adult tissues, semiquantitative-RT-PCR was first performed using *β-actin* as an internal control. The results showed that *Popou5f3* mRNA transcripts were exclusively restricted to the gonadal tissues, but absent in any somatic organs examined, such as the heart, liver, spleen, kidney, brain, intestine, muscle and gill (Figure 4C). Additionally, strong expression was observed in the ovary while a faint band was detectable in the testis. Then, the expression pattern was quantitated and confirmed by qRT-PCR. The female gonad had a dominantly higher expression level of *Popou5f3* than the male gonad (Figure 4B). Furthermore, *Popou5f3-S* showed about 2−5-fold expression level to *Popou5f3-L*.

To define the cellular localization of *Popou5f3* transcripts and PoPou5f3 protein in ovary and testis, ISH and immunohistochemistry (IHC) analyses on paraffin sections of these organs were performed. ISH results showed that the ovary was in development stage II, which was composed of a large number of oocytes. The expression of *Popou5f3* in oocytes was consistent with its maternal expression profile (Figure 5D). Similar with the cellular localization of zebrafish *pou5f3* in oocytes [33], *Popou5f3* mRNA transcripts were also observed throughout the cytoplasm (Figure 5E). In testis, the hybridization signal was only detected in spermatogonia, with no signal in spermatocytes or spermatids (Figure 5F,G), nor in the negative controls (Figure 5d,f).

**Figure 6:**
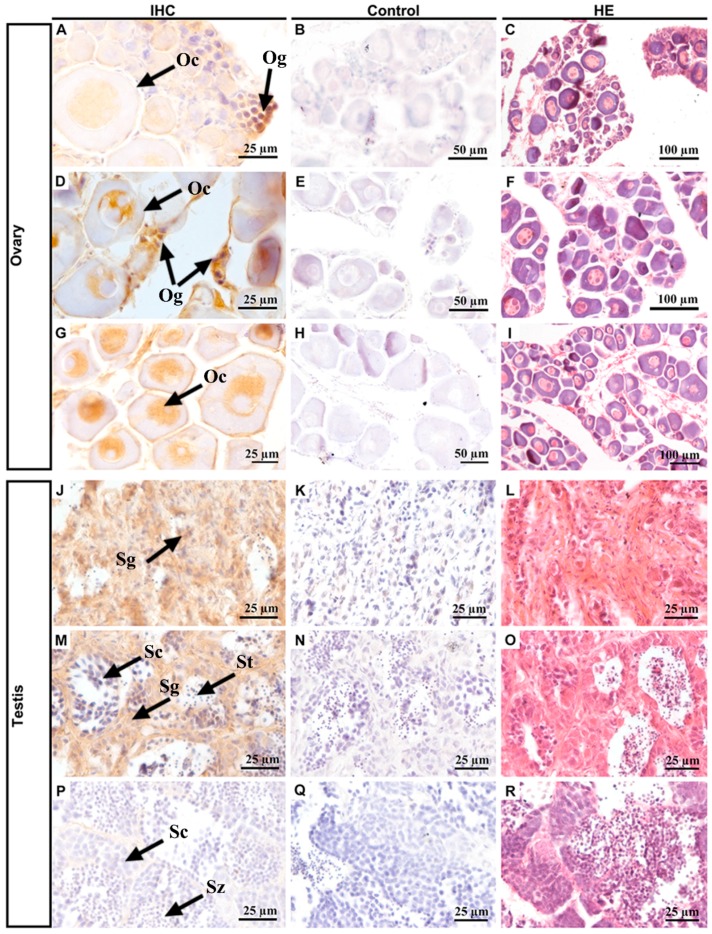
Immnohistochemisiry of localization of *Po*Pou5f3 in *P. olivaceus* ovaries and testes at different stages. Positive signals of anti-*Po*Pou5f3 immunolabeling are shown in tawny (ovaries: (**A**,**D**,**G**); testes: (**J**,**M**,**P**)). Negative controls with the rabbit preimmune serum (ovaries: (**B**,**E**,**H**); testes: (**K**,**N**,**Q**)) and hematoxylin and eosin (HE, ovaries: (**C**,**F**,**I**); testes: (**L**,**O**,**R**)) staining of the corresponding sections were included. Og, oogonia; Oc, oocytes; Sg, spermatogonia; Sc, spermatocytes; St, spermatids; Sz, spermatozoa. Scale bars are indicated in the lower right corner of each photo.

Next, we collected samples at different stages of gonadal development to investigate the cellular localization of *Po*Pou5f3 (Figure 6). The initial observation data and subsequent hematoxylin and eosin (HE) staining showed that the ovaries in panels A–C were 1 ± 0.1 cm long, and had cylindrical rods and a certain number of oogonia and primary oocytes. The ovaries in panels D–F were 2.5 ± 0.5 cm long, triangularly shaped, containing a small number of oogonia and many oocytes. The ovaries in panels G–I were 4.0 ± 0.5 cm long, bearing a slender trident shape, with a large number of primary oocytes. The testes in panels J–L were 0.5 ± 0.1 cm long, resembled thin strips, and were mainly composed of spermatogia with a large size and small nuclear. The testes in panels M–O were 2.0 ± 0.5 cm long, thin strips, and contained a lot of spermatogonia, primary spermatocytes and spermatids. The testes in panels P–R were 5.0 ± 0.5 cm long, of the fleshy lobular type, with plenty of spermatocytes, spermatids and spermatozoa. When compared with those of spermatogonia, the size of primary spermatocytes decreased and the nucleocytoplasmic ratio increased. IHC results showed that *Po*Pou5f3 is located in the nucleus of oogonia and oocytes, especially with a strong signal in oogonia (Figure 6A,D,J). But in the testis, the *Po*Pou5f3 signal was enriched in spermatogonia and became undetectable when spermatogenesis proceeded (Figure 6J,M,P). Taken together, these results indicate that the expression of *Popou5f3* is specific to gonadal tissues and it peaks in oogonia and spermatogonia.

## 3. Discussion

*Pou5f1* is an important marker gene of stem cells. In the past 20 years, the homologues of *Pou5f1* in mammals as well as in other vertebrates, including human [34], bovine [35], brushtail possum [36,37], rhesus monkey [38], platypus [39], vole [40], chicken [41], xenopus [42], zebrafish [21,43], medaka [17,44], Chinese sturgeon [45], goldfish [18], and Nile tilapia [19], have been identified. Several research studies have revealed their critical roles during early embryonic development and their importance in the maintenance of stem cell pluripotency. In this study, we have cloned and characterized *Popou5f3* from the marine fish Japanese flounder, analyzed its sequence features and expression patterns, which lays a foundation for further functional study on stem cell tracing and manipulations in this species.

### 3.1. Popou5f3 Is the Homologue of Mammalian POU5F1

Previously, the names of the homologues of mouse *Pou5f1*/*Oct4* in teleost fish were not unified, such as the originally named *pou2*/*spg*, then *pou5f1* in zebrafish [20,21,46], *oct4* in medaka and annual killifish [17,44,47], *pou2* in *Gadus morhua*, Chinese sturgeon, and goldfish [18,30,45], which creates confusion in their naming. However, sequence and synteny conservation analyses indicate that zebrafish *pou2* is not the true orthologue of mammalian *POU5F1*, but is closely related to mammalian *POU5F3*. Due to this reason, the zebrafish nomenclature committee renamed it zebrafish *pou5f3* [48,49]. In addition, scholars recommend the application of this nomenclature to all vertebrate orthologues of *POU5F3* [49,50].

In our study, we have cloned the complete sequence of *pou5f3* in Japanese flounder and proven it to be the homologue of mammalian *POU5F1*. In the studies about the origin of *POU5F1*, it is suggested that the two copies of *POU5F1* and *POU2* (now called *POU5F3*) existing in marsupials and monotremes originated from the *Pou2* in lower vertebrates [39,48,49]. Furthermore, *POU5F1* is only present in eutherian mammals; however, there are many *POU5F1* pseudogenes [51]. Indubitably, complex evolutionary events, such as gene duplication, loss, chromosome rearrangement, etc., have occurred in the process of *POU5F1*/*POU5F3* evolution. In our study, we found only one gene copy of *Popou5f3* via genomic sequence searching, which is coincident with other identified teleost *pou5f3* [19,44]. Moreover, *Popou5f3* has a conserved chromosome synteny and a high sequence similarity with medaka and zebrafish *pou5f3*. Therefore, we conclude that *Popou5f3* is the orthologue of teleost *pou5f3*.The protein encoded by *Popou5f3* has a characteristic POU domain, which shares high sequence identities over 68.5%, with other representative species and it has a similar three-dimensional structure to the POU domain of mouse Pou5f1 (Figure S3). Among different species, *Pou5f1*/*pou5f3* have the same genomic organization composed of five exons and four introns, and the same positions of the POU domain in the gene [32,52]. All these indicate that the POU domain is primarily conserved in evolutionary development. Studies have demonstrated that the POU domains of mammalian Pou5f1 as well as zebrafish Pou5f3 can bind to the octamer motif containing a conserved ATGCAAAT sequence and activate the transcription of the target gene or itself [52]. For Pou5f1, it can further interact with the HMG domain of Sox2 to form a complex to regulate the pluripotency maintenance of stem cells [4,53], which illustrates its functional importance. Based on the sequence and structural similarity, we speculate that *Po*pou5f3 may have similar functions as other species. However, the extent of conserved functions of *Po*Pou5f3 still needs further investigation.

### 3.2. The Potential Cis-Regulatory Elements (CREs) in Regulating Popou5f3 Expression

CRE regions of DNA typically regulate the nearby gene expression by functioning as binding sites for transcription factors and may be located either at 5’ or 3’ to the coding sequence of the gene. As vital components of genetic regulatory networks, CREs also have an important evolutionary role [54,55]. Thus, it is essential to characterize the regulatory regions of *Popou5f3* for a better understanding of the regulation of its expression and function. For this purpose, we first obtained the flanking sequences of *Popou5f3* and analyzed these sequences with a number of potential *cis*-elements for TF binding. In mice, the transcriptional initiation of *Oct4*/*Pou5f1* is regulated by a germ cell (GC)-rich proximal promoter sequence that lacks the TATA-box element, and its expression is also regulated by the proximal and distal enhancer elements within the upstream region [56]. Moreover, a variety of TFs can be combined with the corresponding *cis*-elements on human *Oct4* promoter region to regulate its gene expression [57]. For example, in retinoic acid-induced stem cells, transcription Specificity Protein 1 (Sp1) regulates *Oct3*/*4* gene expression by binding to the corresponding locus on the promoter, which results in stem cell differentiation [58]. The germ cell nuclear factor (GCNF) can also be combined with the corresponding site on the promoter to induce gene silencing [59]. In this study, we cloned the complete *Popou5f3* promoter sequence through two rounds of chromosome walking. Consistent with the report in mice [56], we also did not find traditional TATA box element in *Popou5f3*. Nevertheless, the transcription factors and related signal pathways involved in regulating stem cell pluripotency, such as FOXP1-ES, LEF1/TCF, PRDM14, STAT, etc. [60,61,62,63,64], were predicted to be abundant in the regulatory region of the gene. In the mammalian *Oct4* gene promoters, there are four conserved regions (CR1-CR4), which are involved in the regulation of gene expression [56,65,66]. However, the promoter sequence of *pou5f3* in teleost, such as flounder, stickleback, tilapia and zebrafish, is less conservative when compared to those of mammals, as it has only one region sharing some similarity with mammalian CR2. Besides, three additional conserved regions and potential conserved *cis*-elements among different species were identified, which suggests that these sequences may be involved in regulating the transcription and expression of *Popou5f3*. In zebrafish, Pou5f3 has been demonstrated to be able to bind to the octamer sequences in upstream DNA to positively regulate its expression through an autoregulatory loop [52]. This provides a worthy place to begin exploring the regulation mechanism for *Popou5f3* since we have also identified potential Oct binding sites in the upstream region. On the other hand, through in vitro experiments, Hong et al. [67] have found that the mouse *Oct4* gene promoter can be activated in the medaka embryonic stem cells. This indicates that some of the *cis*-elements and regulatory sequences involved in regulation of the pluripotency specific gene expression are conserved between mammals and teleost fish. Actually, this part of the results is only from bioanalytical level analysis and prediction. Thus, we need further experiments in order to verify whether it has a regulatory function.

It is well known that different spliced variants of *pou5f3*/*OCT4* had been identified and their functionality with distinct capacities was analyzed both in mammals and teleost [21,68,69]. However, we did not find any alternative splicing in *Popou5f3*. Interestingly, we have found the presence of APA, and it generated two kinds of *Popou5f3* transcripts with different 3’-UTR length, which have not been reported in this gene of other species yet. Recently, a great deal of attention has been paid to APA due to its central role in post-transcriptional gene regulation, modulation of genes that are critical for stem cell function, development and disease [70,71,72]. Mechanically, changes of binding sites for RNA binding protein or microRNA on 3′-UTR can affect the stability and localization as well as the translation efficiency of mRNA. The cleavage and polyadenylation specificity factor (CPSF)-associated protein, Fip1, is able to activate the ESC-specific APA profiles to ensure the optimal expression of self-renewal genes [73]. Moreover, the APA-mediated shorter 3′-UTR shift of key myogenic factor, Pax3, can render itself resistant to regulation by miR-206, thus maintaining the muscle stem cell function [72]. With regard to *Popou5f3*, multiple *cis*-elements for mRNA 3′-processing proteins such as PTB, AUBF, CtsF, and CPE were identified, which might be involved in post-transcriptional regulation of *Popou5f3*. Nowadays, the mechanism and regulation of non-coding RNAs, especially microRNAs, have been extensively and deeply investigated in mammals, whereas study in teleost fish is still in its infancy stage. Here, we provide new ideas and directions for future study.

### 3.3. Popou5f3 Is Abundant at Blastula-Stage Embryos and Restricted in Adult Gonads

As the expression of *Pou5f1* mRNA is closely related to the cell fates, the *Pou5f1* homologue has been regarded as a master regulator of self-renewal in pluripotent stem cells as well as cell reprogramming in mammals [23,50,74]. Nevertheless, a considerable difference in expression pattern of *pou5f3* exists among lower vertebrates. In zebrafish, *pou5f3* was maternally expressed, increased to reach its peak at the gastrula stage, restricted in the neural plate and afterwards confined to the caudal end of the spinal cord, showing vital roles during development, such as brain regionalization, mesendoderm specification and endoderm formation [20,21,43,75]. Certain similarity was found in Nile tilapia *pou5f3*, in that it is expressed until the blastula stage and transiently appears in the brain region before hatching [19]. On the other hand, the Atlantic cod *pou5f3* (originally called *ac*-*Pou2*) showed high expression levels at early development stages before gastrulation as well as the ESC isolated from blastula stage eggs, which illustrates its role in marking the undifferentiated blastula cells in vivo and in vitro [30]. The same expression profiles of medaka *pou5f3* during embryogenesis coupled with functional verification demonstrated thoroughly that it is essential for pluripotency maintenance [17,44]. For adults, though we did not observe its expression in brain tissue, the mRNA and protein distribution of *Popou5f3* are similar with those of medaka *pou5f3* in gonads, which is specific to germ cells. Moreover, *Popou5f3* was maternally inherited, abundantly expressed at the blastula stage, then gradually diminished to barely detectable by the end of the gastrula stage, a period in which cells are differentiated into three embryonic germ layers. Generally, *Popou5f3* showed a more similar expression pattern with medaka rather than zebrafish. Combined with our previous findings on another two pluripotent-associated genes (*Ponanog* and *Posox2*), it appears that these three genes might form an interactive network to regulate the pluripotent state of blastula cells and subsequent germ layer differentiation in *P. olivaceus* [31,76,77]. Currently, we are focusing our next studies on finding whether *Popou5f3* is expressed in primordial germ cells and the functions of the two different-length transcripts of *Popou5f3*.

## 4. Experimental Section

### 4.1. Animals and Handling

The Japanese flounder (*P. olivaceus*) used in this study was sampled from local mariculture farms in Haiyang City, China. All handling of the samples were conducted and approved by the Institutional Animal Care and Use Committee of the Ocean University of China and the China Government Principles for the Utilization and Care of Vertebrate Animals Used in Testing, Research and Training (State Science and Technology Commission of the People’s Republic of China for No. 2, 31 October 1988).

Fertilized eggs were obtained by artificial fertilization and they were incubated in clean sea water with continuous aeration at 17 ± 1 °C. During the development stage, embryos were observed under a microscope and three pools of embryos that came from mixed families were collected separately at a particular stage [78]. There were 16embryonic stages (unfertilized egg (UF, 0 h), one-cell (0.5 h after fertilization (haf)), two-cell (1.8 haf), eight-cell (3.5 haf), 16-cell (4.6 haf), 32-cell (5.3 haf), multi-cells (10.3 haf), morula (10.6 haf), high blastula (17 haf), low blastula (17.5 haf), early gastrula (20.1 haf), mid-gastrula (25.6 haf), late gastrula (28.5 haf), neurula (30.6 haf), heart-beating (67.1 haf), and hatching stage (93 haf)) that were selected for this study. The embryos were sampled by immersion in 1 mL RNAwait liquid (Solarbio, Beijing, China), and then immediately stored at −80 °C until use. Tissue samples of the heart, liver, spleen, kidney, brain, intestine, muscle, gill, and gonads (ovary and testis) were collected from six randomly selected healthy adults (three females and three males), immediately frozen in liquid nitrogen, and stored at −80 °C until use. Embryos (at eight-cell, high blastula, and tail-bud stages (42 haf)) and gonad (ovary and testis) samples used for ISH were immediately fixed in 4% paraformaldehyde (PFA)−phosphate buffered saline (PBS) (4% PFA overnight at 4 °C, dehydrated in a gradient-increasing methanol, and stored in 100% methanol at −20 °C. The embryos were microscopically dissected to remove the envelopes before dehydration. Gonads used for immunohistochemistry were collected from 4-, 10- and 16-month-old fish samples, respectively, and fixed by immersion in fresh Bouin′s fixative for 20 h, then dehydrated and stored in 100% ethanol.

### 4.2. Genomic DNA, Total RNA Isolation and cDNA Synthesis

Genomic DNA was extracted from the muscle tissue via the phenol-chloroform procedure. Total RNA was isolated separately from the sampled embryos and tissues using Trizol reagent (Invitrogen, CA, USA) according to the manufacturer’s protocol and treated with RNase-free DNase I (Takara, Dalian, China) to remove contaminating genomic DNA and stored at −80 °C. The quantity and quality of genomic DNA and total RNA were determined by agarose gel electrophoresis and Nanophotometer Pearl (Implen GmbH, Munich, Germany). The first-strand cDNA was synthesized using M-MLV reverse transcriptase (Takara, Dalian, China) as described in the protocol. The corresponding cDNA was stored at −20 °C.

### 4.3. Cloning and Sequencing of Popou5f3

The semi-nested degenerate primers, pou5f3-core-Fw and pou5f3-core-Rv-1/2 (Table 2) were used to amplify *Popou5f3* cDNA fragment. They were designed according to the evolutionary conserved domains of *pou5f3* in other fish species obtained from the NCBI. The untranslated regions of *Popou5f3* cDNA were obtained by 5′ and 3′ RACE with the SMART™ RACE cDNA Amplification kit (Clontech, CA, USA) and by using the gene-specific primers 5’-RACE-pou5f3 and 3′-RACE-pou5f3 (Table 2). The genomic DNA sequence were obtained by primers pou5f3-full-length-Fw/Rv (Table 2). The promoter region was amplified by two rounds of PCR strategy using six gene-specific primers (Table 2, pou5f3-Pro-SP1/2/3-1st and pou5f3-Pro-SP1/2/3-2nd) and four shorter arbitrary degenerates (AP1/2/3/4) supplied with the Genome Walking kit (Takara, Dalian, China) under the manufacturer’s instructions.

### 4.4. Sequence Analysis

The exon and intron boundaries were determined by alignment of the obtained cDNA sequence with the generated genome sequence. NCBI′s online ORF Finder and DNASTAR were employed to predict ORF for translated peptide product. The POU domains were identified using the simple modular architecture research tool (SMART) and InterProScan search software. The multiple alignments of Pou5f3 were generated with ClustalW and the phylogenetic tree was constructed using the MEGA 5.2 program by the neighbor-joining method with 1000 bootstrap replicates. Bioinformatic analysis and potential transcription factor binding sites within the 5′ regulatory region of *Popou5f3* was performed with online program MatInspector. The conserved promoter sequences between fish and mammals were analyzed by the MEME software and the Dialign software from the Genomarix suite. The websites of the software mentioned above were listed in Table 3.

### 4.5. Quantitative Expression Analysis of Popou5f3

Semiquantitative-RT-PCR was carried out to determine the tissue distribution of *Popou5f3* using *β-actin* as an endogenous reference. The primers, pou5f3-SRT-Fw and pou5f3-SRT-Rv (Table 2), located in the exon 1 and exon 2, respectively, with a 605 bp of product, were used for semi-RT-PCR. qRT-PCR was used to quantify the expression levels of *Popou5f3* at different developmental stages. The differences between the two sexes were identified using Light-Cycler Roche 480 (Roche Applied Science, Mannheim, Germany) according to the method we described previously [31,79]. Since we had found two transcript isoforms but could not design appropriate primers to distinguish them exactly, we used primers pou5f3-RT-Fw/Rv and pou5f3-L-RT-Fw/Rv (Table 2) for qRT-PCR to amplify the total *pou5f3* and *pou5f3-L*. These two primer pairs are located within exon 1 and the *pou5f3-L* specific 3′-UTR region, respectively.

### 4.6. In Situ Hybridization Analysis

Digoxigenin-labelled RNA sense and antisense probes were synthesized using a DIG RNA labelling kit (Roche, Mannheim, Germany) with pou5f3-ISH-Fw/Rv (Table 2) specific primers spanning the partial 5′-UTR and N-terminal coding region of *Popou5f3* cDNA. RNA whole-mount ISH for embryos and section ISH for gonadal tissues were performed as previously described [31,79].

### 4.7. Immunohistochemistry and Histological Analysis

Full-length cDNA fragment encoding 475 amino acids of PoPou5f3 was fused to the His tag by insertion between the *Eco*RI-*Not*I sites in pET-32a (+) (Novagen, Darmstadt, Germany). The recombinant fusion protein was expressed in *E. coli* Transetta (DE3) (Transgene, Beijing, China) and purified using the Ni-NTA His·Bind^®^ Resins (Novagen, Darmstadt, Germany) according to the manufacturer′s protocol. Purified protein was sent to Shanghai Shenggong Biological Engineering Co., Ltd. (Shanghai, China) for the production of the polyclonal antibody against PoPou5f3. The antiserum was stored in aliquot at −20 °C. The immunohistochemical staining for Pou5f3 was performed using a standard indirect peroxidase method. Briefly, the dewaxed and rehydrated sections were incubated with 3% H_2_O_2_ at room temperature for 15 min to inhibit the endogenous peroxidase. Subsequently, the sections were heated in ethylenediaminetetraacetic acid (EDTA) buffer with an oven at 85 °C for 40 min to unmask the antigen, followed by blocking with 2% non-fat milk at room temperature for 2 h. The incubation with the primary anti-Pou5f3 antibody with a dilution of 1:500 was performed overnight at 4 °C. Pre-immune rabbit serum was used for the negative control group. Subsequently, the secondary horseradish peroxidase (HRP)-conjugated goat anti-rabbit IgG antibody (1:5000) was applied, and then visualized using 3,3′-diaminobenzidine (DAB). Between all steps the sections were thoroughly washed with PBST. Finally, sections were lightly counterstained with haematoxylin and mounted in resin. For histological analysis, sections were stained with Hematoxylin and eosin (HE). Nikon Eclipse Ti-U microscope (Nikon, Tokyo, Japan) was used to observe and photograph the results.

### 4.8. Statistical Analysis

All data were represented as mean ± standard error of the mean (SEM). Statistical differences were calculated using one-way ANOVA followed by Duncan′s test with SPSS 20.0 software. *p* < 0.05 were considered statistically significant.

## 5. Conclusions

In conclusion, we have identified and characterized the structure and expression profiles of *pou5f3* in *P. olivaceus*. Evidence has been gathered from gene structure, chromosome synteny and phylogenetic position and prove that *Popou5f3* is the homologue of mammalian *Pou5f1*. Plenty of *cis*-regulatory elements are predicted in the flanking regions, which could be involved in the regulation of *Popou5f3* expression. A very high and specific expression level of *Popou5f3* at blastula stage during embryonic development and adult germ stem cells suggests its potential roles in lineage formation and pluripotency maintenance. Significantly, this is the first report showing that *Popou5f3* has two transcripts with different lengths of 3′-UTR generated by alternative polyadenylation (APA). These findings may extend the understanding of the function and evolution of *pou5f3* in lower vertebrates. Moreover, our study provides fundamental information for stem cell tracing and genetic manipulation in *Paralichthys olivaceus*.

## Figures and Tables

**Table 1:** Identity between POU (Pit-Oct-Unc) V proteins.

*Po*Pou5f3 Vs *	Full-Length (%)	N-Terminal (%)	POU Domain (%)	POU Domain			C-Terminal (%)
				POUs (%)	Linker (%)	POUh (%)	
*H.s* POU5F1	37.9	23.4	68.5	75.7	36.8	69.6	14.3
*B.t* Pou5f1	39.3	20.4	68.5	74.3	36.8	71.4	15.9
*M.m* Pou5f1	39.4	20.0	68.5	73.0	36.8	73.2	14.5
*T.g* Pou5f3	49.5	10.8	74.2	87.8	40.0	68.4	32.3
*A.c* Pou5f3	37.2	15.7	69.6	81.1	22.2	69.6	14.5
*A.m* Pou5f3	43.4	19.1	73.5	89.2	25.0	70.2	41.2
*T.r* Pou5f3	81.5	75.1	94.7	94.6	85.0	98.2	76.1
*O.l* Pou5f3	80.8	72.5	97.4	98.6	90.0	98.2	74.3
*D.r* Pou5f3	70.4	58.4	94.7	95.9	90.0	94.7	60.0

* Percentage of amino acids identity between indicated domains of *Po*Pou5f3 and other vertebrate POU V proteins. Abbreviations: *Po*, *Paralichthys olivaceus*; *H.s*, *Homo sapiens*; *B.t*, *Bos taurus*; *M.m*, *Mus musculus*; *T.g*, *Taeniopygia guttata*; *A.c*, *Anolis carolinensis*; *A.m*, *Ambystoma mexicanum*; *T.r*, *Takifugu rubripes*; *O.l*, *Oryzias latipes*; *D.r*, *Danio rerio*.

**Table 2:** Sequences of the primers used in this study.

Experiment	Primer Name	Primer Sequences (5′–3′)
Core fragment	pou5f3-core-Fw	TGTTCAGYCAGACVACMATY
	pou5f3-core-Rv-1	CDCGBACYACRTCTCTCTC
	pou5f3-core-Rv-2	AGCAGYGGGTCVTCCAG
RACE PCR	5′-RACE-pou5f3	GTAATCTCCTGGGTGTTGGGTTTG
	3′-RACE-pou5f3	GCAGAGACCTCAGACAATCCCCAG
Full-length cDNA	pou5f3-full-length-Fw	CTATCTCGGACTTGTTCTTG
	pou5f3-full-length-Rv	CATAACTTGCCTTTGCTG
Genome walking(1st)	pou5f3-Pro-SP1-1st	CTTCCAAACGCTGTGGGAGATT
	pou5f3-Pro-SP2-1st	CAAAGAGGAACGAGCGGAGGAC
	pou5f3-Pro-SP3-1st	TGCCACATTACCACCAACACGC
Genome walking	pou5f3-Pro-SP1-2nd	TATCGTGCCCTTATGGTGTCCTT
	pou5f3-Pro-SP2-2nd	TAAAGCACCCATGTAGGTCAA
	pou5f3-Pro-SP3-2nd	CTCAGCCATGCAAGCATTGTCC
In Situ hybridization	pou5f3-ISH-Fw	ACAGGTCGACTGAAACGGATGAGCACG
	pou5f3-ISH-Rv	AGCGGAATTCGTGATGCCACCATACGC
Prokaryotic expression	pou5f3-PE-Fw	CGAATTCATGTCTGAAAGATCTCAGAGTC
	pou5f3-PE-Rv	TGCGGCCGCTTATCCAGTCATGTGACCAAT
SemiRT-PCR	pou5f3-SRT-Fw	AACACTGGTATCCCTTCGCT
	pou5f3-SRT-Rv	CATCTTACCATAGAGGTTACCC
Reference gene	β-actin-Fw	GAGATGAAGCCCAGAGCAAGAG
	β-actin-Rv	CAGCTGTGGTGGTGAAGGAGTAG
qRT-PCR	pou5f3-RT-Fw	GAGGCGTGTTGGTGGTAATGTG
	pou5f3-RT-Rv	AAGAGGAACGAGCGGAGGAC
qRT-PCR	pou5f3-L-RT-Fw	TGACTGTAACCACCTGAGACCTAA
	pou5f3-L-RT-Rv	AAGATTTACTGGGAAAAGGAACAA
qRT-PCR	18S-RT-Fw	GGTAACGGGGAATCAGGGT
	18S-RT-Rv	TGCCTTCCTTGGATGTGGT
qRT-PCR	UbcE-RT-Fw	TTACTGTCCATTTCCCCACTGAC
	UbcE-RT-Rv	GACCACTGCGACCTCAAGATG

**Table 3:** The websites of the online software used in this study.

Website Name	Link
NCBI′s online ORF Finder	http://www.ncbi.nlm.nih.gov/gorf/gorf.html
SMART	http://smart.embl-heidelberg.de/
InterProScan	http://www.ebi.ac.uk/Tools/pfa/iprscan/
MatInspector	http://www.genomatix.de/matinspector.html
MEME	http://meme.nbcr.net/meme/cgi-bin/meme.cgi
Genomarix suite	http://www.genomatix.de/cgi-bin/dialign/dialign.pl

## Supplementary Materials

Supplementary materials can be found at www.mdpi.com/1422-0067/18/1/231/s1.
