# Supplementary material for: Evolutionary Conservation of pou5f3 Genomic Organization and Its Dynamic Distribution during Embryogenesis and in Adult Gonads in Japanese Flounder Paralichthys olivaceus
Source: Int J Mol Sci. 2017 Jan 23;18(1):231. doi: 10.3390/ijms18010231 (PMC5297860; doi:10.3390/ijms18010231)
Supplement: Supplementary file 1 [file ijms-18-00231-s001.pdf]

Supplementary Materials: Evolutionary Conservation of *pou5f3* Genomic Organization and Its Dynamic Distribution during Embryogenesis and in Adult Gonads in Japanese Flounder *Paralichthys olivaceus*

Jinning Gao, Quanqi Zhang and Xubo Wang

Table S1. The Genbank accession numbers or Ensembl IDs of the POUV proteins analyzed in Figure 1C.

Species	Genbank Accession No./ Ensembl IDs	Species	Genbank Accession No./ Ensembl IDs
Acipenser sinensis	AEK81554.1	*Pan troglodytes*	NP_001238970.1
Anolis carolinensis	ENSACAP00000017001	*Ornithorhynchus anatinus*	NP_001229656.1
Bos taurus	NP_777005.1	*Oryzias latipes*	NP_001098339.1
Carassius auratus	AET79963.1	*Oreochromis niloticus*	XP_003444455.1
Danio rerio	NP_571187.1	*Pagrus major*	BAH08689.1
Gadus morhua	ENSGMOP00000005053	*Paralichthys olivaceus*	KJ522774
Gasterosteus aculeatus	ENSGACP00000023125	*Rattus norvegicus*	NP_001009178.1
Homo sapiens	NP_002692.2	*Takifugu rubripes*	XP_003965650.1
Labeo rohita	ADC96616.1	*Tetraodon nigroviridis*	ENSTNIP00000016222
Latimeria chalumnae	XP_005994419.1	*Xenopus tropicalis*	ENSXETP00000008656
Mus musculus	NP_038661.2	*Xiphophorus maculatus*	XP_005799711.1

Table S2. The Ensembl IDs of the analyzed sequences in Figure 3B.

Species		Ensembl IDs
Human	*Homo sapiens*	ENST00000259915
Cow	*Bos taurus*	ENSBTAT00000028122
Mouse	*Mus musculus*	ENSMUST00000025271
Stickleback	*Gasterosteus aculeatus*	ENSGACT00000023169
Tilapia	*Oreochromis niloticus*	ENSONIT00000004857
Zebrafish	*Danio rerio*	ENSDART00000065817

```
gtgcccacgaaaacctgtggagccttggaggagagcagcatttgagcttgacggtggagcggcgtgaagggcaaggattcatggagtaggccggaggcgt  100
gttggtggtaatgtggcaccaaaacgacagctgctaaggccttttgcaaaactctccccaaacggactttacgcaagcaaacagttgatggaattttgga  200
gcgcgtcctccgctcgttcctctttgacttgtgcgtaaacttgaaacggatgagcacgtttaactttattcccattaactatttcttttaaaactttcta  300
tctcggacttgttcttgttaaataatctcccacagcgtttggaagATGTCTGAAAGATCTCAGAGTCCGGAGTGCCAAAGCAGGCCGTATGACTTCAGCC  400
                                              M  S  E  R  S  Q  S  P  E  C  Q  S  R  P  Y  D  F  S   18
GCGCCGGCCATTGCGCTCAGGTTTTGGGTCAAGATGGTTTGGGCAACGCTGCGTCATTCCAGCTTCCTCACGGCGTTTTGCCAGACCCGAGCCTCCTCTA  500
R  A  G  H  C  A  Q  V  L  G  Q  D  G  L  G  N  A  A  S  F  Q  L  P  H  G  V  L  P  D  P  S  L  L  Y   52
TAACAAAACGGCGTATGGTGGCATCACGTCGGCCTCTGCGCAGACTTTCTTCCCATTCCCACCCATGGCCAGTGACTACAGGGGAACCGACCTGCAGGCT  600
 N  K  T  A  Y  G  G  I  T  S  A  S  A  Q  T  F  F  P  F  P  P  M  A  S  D  Y  R  G  T  D  L  Q  A   85
GGGGAGTTTGGGCAACCCAAACACTGGTATCCCTTCGCTGCGCCCGAGTACACCGGCCAGGTACCCGGCGTAACAGCAGCTACCCAGCCTACTAACCTGA  700
G  E  F  G  Q  P  K  H  W  Y  P  F  A  A  P  E  Y  T  G  Q  V  P  G  V  T  A  A  T  Q  P  T  N  L    118
GTCCCCCTATGGCCGAGACCCGGGAGCAAATCAAAATGCCCGAGATCAAAACCGAGAAGGACACCGGCGATGACTACTCAACGGAGATCAAGGGTCAACA  800
S  P  P  M  A  E  T  R  E  Q  I  K  M  P  E  I  K  T  E  K  D  T  G  D  D  Y  S  T  E  I  K  G  Q  Q  152
GTATCTGACACCGTCAGCCTCCGCCGCCATGGCTCATGGAGTCTTCTACCCCGCGGCCTGGAACCCGTCCTTCTGGCCTGGAATCGCCCACATCACCCCG  900
 Y  L  T  P  S  A  S  A  A  M  A  H  G  V  F  Y  H  A  A  W  N  P  S  F  W  P  G  I  A  H  I  T  P  185
CCCGGTATCAGTAATCAAAATCCTTCCACATCCTCTGCATCGTCGCCGTCTATGTCCCCTTCACCCCCGAGCAACGGGCTTCCAGGAAACGCGTTTTTCA  1000
P  G  I  S  N  Q  N  P  S  T  S  S  A  S  S  P  S  M  S  P  S  P  P  S  N  G  L  P  G  N  A  F  F   218
GCGTGAACCCAACCCAAGCCGCCCCCGGGCCGCAGACGCAGAACCCGGCCTCCTCCACGCGGAGCAGCGGTTCCTCCAGCGGCGGATGCAGCGACTCAGA  1100
S  V  N  P  T  Q  A  A  P  G  P  Q  T  Q  N  P  A  S  S  T  R  S  S  G  S  S  S  G  G  C  S  D  S  E  252
GGAGgtaagattagaatcccttcaaccaaccatttaatccaacagttgtaatatgagccttcaagtcaaacacacatgtgttcaacttctgtaagttcta  1200
 E                                                                                                   253
catctatatggttttcattttcaacctgtctcacagGAGAACCTCTCCACTGAGGAACTGGAGCAGTTTGCCAAGGAACTGAAACACAAACGCATCACTT  1300
                                        E  N  L  S  T  E  E  L  E [Q  F  A  K  E  L  K  H  K  R  I  T]  274
TGGGTTTCACTCAAGCTGATGTTGGCCTTGCCCTGGGTAACCTCTATGgtaagtttggttcccatgtggaaacgtcctattgggtcatattgcactccct  1400
[L  G  F  T  Q  A  D  V  G  L  A  L  G  N  L  Y]                                                     290
ccctaatgctttcaaagattcttatttgaagtaataatgatggactttctagtattggttttgtatgttgcacagttcagattcttatctacgtcagtag  1500
aaggttacttgttatgttttgtgtgttgaggcaggaggtcactggtttggcaaagtatctctgattgccactcctgaaatcttgctgaatcatcagccat  1600
ccaatgtgttgagattttggacagatacatgttgcatgagtcttgtctattcagagataatggcatgtgaaatctgatgatttatatttgcgtgcagGT  1700
                                                                                                  [G]  291
AAGATGTTCAGCCAGACGACCATTTGCCGCTTCGAGGCTCTGCAGCTGAGCTTTAAGAACATGTGCAAGCTGAAACCCCTTCTTCAGAGATGGCTGAATG  1800
[K  M  F  S  Q  T  T  I  C  R  F  E  A  L  Q  L  S  F  K  N  M  C  K  L  K  P  L  L  Q  R  W  L  N]  324
AGGCAGAGACCTCAGACAATCCCCAGGATgtaagtctgcaacactgacatctgccagctagtttacaagacaacagatgatccttgagcctgatgggatc  1900
[E  A  E] T  S  D  N  P  Q  D                                                                        334
agttggtcttttcagactgagctgactttaaccaaagcactttgttctcaggaagtgtgacctgccatctagtggcaacattaatttggtctctttagag  2000
atcacatgaaacattatttgaaccataaccatccatacaaggcttaaaccaaaagggacacaaattgcttcggtacataggtagtcctagttggggcttg  2100
tcataccttttttgtacatcttacatattctcaaattatgtgttattcctcagATGTACAAGATCGAGAGAGTGTTTGTTGACACCAGAAAGAGGAAGAG  2200
                                                     M  Y  K  I  E  R  V  F  V  D  T  R [K  R  K  R]  350
AAGGACTAGTCTGGAGGGGGCAGTGCGCTCTGCTCTGGAGTCCTACTTTATCAAGTGTCCCAAACCCAACACCCAGGAGATTACACACATTTCAGATGAC  2300
[R  T  S  L  E  G  A  V  R  S  A  L  E  S  Y  F  I  K  C  P  K  P  N  T  Q  E  I  T  H  I  S  D  D]  383
CTGGGCCTTGAGAGAGACgtaagaacaaaagtgacgtagtttgccttctaaagtatgtgtcttgttgtggttgtaaacgagaaatcttgttcttggcagG  2400
[L  G  L  E  R  D]                                                                                    389
TGGTGCGTGTTTGGTTCTGCAACCGGAGACAGAAAGGAAAACGACTGGCTTTGCCACTGGATGAGGAGTGCGACGGCCAGTATTACGAGCAGAGCCCTTC  2500
[V  V  R  V  W  F  C  N  R  R  Q  K  G  K  R] L  A  L  P  L  D  E  E  C  D  G  Q  Y  Y  E  Q  S  P  S  423
CCCACTGAACATGGCCCCTTCCCCCATCCCCAGTCAGAGCTACCCTGTGTCCAGCTACCCCGGAGCCACTCCTACCACACTGTACATGCCCCCCCTTCAC  2600
 P  L  N  M  A  P  S  P  I  P  S  Q  S  Y  P  V  S  S  Y  P  G  A  T  P  T  T  L  Y  M  P  P  L  H  456
CGGCCCGATGTCCTGAAACAGGCCCTTCACCCCGGACTTATTGGTCACATGACTGGATAAacacgctccggttggcctagccaatcccgcagataacctt  2700
 R  P  D  V  L  K  Q  A  L  H  P  G  L  I  G  H  M  T  G  *                                         475
cccaagcttttctgtcaaaggccaactcaaactggagagggacgatcagtggatcacaggacaaaccgttcaagggaggaggatctttttcctgttattc  2800
tgaaactacacaaattgcattgtccacatcctctaaccatgaccagaaactccacaccaacaattttggatcaagtcttggattaagcatagtaaatcaa  2900
tccactggctttattcagttcatgtttagaacaatttaagatttagctttgtacagcaaaggcaagttatggaatgatgtaaagaacctgaacgttcctt  3000
ccacttatactaaatcctggtaggttttctggggtgcaggtgagaacaggtctaggttatcaagagaaccaaaatgtgagtaatagaattattttttta  3100
gagttgggtcagtattttgtgccatacttgtcaccaatctgaaaagagcagctacggtatttgtcttccatttgaaatggagaacagtttcttattgggg  3200
ttaatttttttccatattattttgcacaaagttgcttttgtaatgttgaacattcttttcaaaaatgttcccgtttgtttaccaaaccaatttttttgtc  3300
ttatagttgttggttgtcatcgacatcttgagcaagtatttttggcagcgatggagggattcaaagtaatattttgatgaaatgtgaagaatcttttgtt  3400
cagtgtcttagctgctaaatctgccatttgcactctgtcaatattttctcttcatgtctcaggtgagtggcagtgagataaatatccgacctgactcagc  3500
tatcgccaa(aaaaaaaaaaaaaaaaaaaaaaaaa)tgtgactgactgtgttctttattcccaccctattgtaaaggttttatgttaatgttgaaatta  3573
caccgtgtcaagtgctcactgaaatatattttaagaagttgcttttagtggaatgatcatagttttagaaagaagacatcaaatgtctcattttgcttc  3673
aaaatacctcacatgactgaagtccttaagtgaccatcaaatcaacttggagatgtcacattaacattggattaatctttaacttttaatataacacgtt  3773
gtctcgacagattgtaaatggattcattataatttattgaaatgttggttaagctgagatgttttgactgtaaccacctgagacctaaatgtaaaacgga  3873
cacttttatttgaaaaattgtatatgcaaactaccaaattgttccttttcccagtaaatctttgcacatgtcccaccaaaa(aaaaaaaaaaaaaaaaaa  3973
aaaaaa)                                                                                               3979
```

Figure S1. Nucleotide and protein sequences of Japanese flounder *pou5f3* gene (accession number: KJ522774). Coding sequences (CDS) are denoted in uppercase letters, whereas the UTRs or introns are denoted in lowercase letters. The deduced amino acids are indicated in one-letter code and exhibited below the coding regions. The GT/AG intron/exon boundaries are shown in italic and the start and termination codons are highlighted in bold. The POU-specific domain and POU homeodomain are shadowed with light and dark gray boxes, respectively. The red letters indicate the poly (A) tails of the two isoforms of *Popou5f3* transcripts.

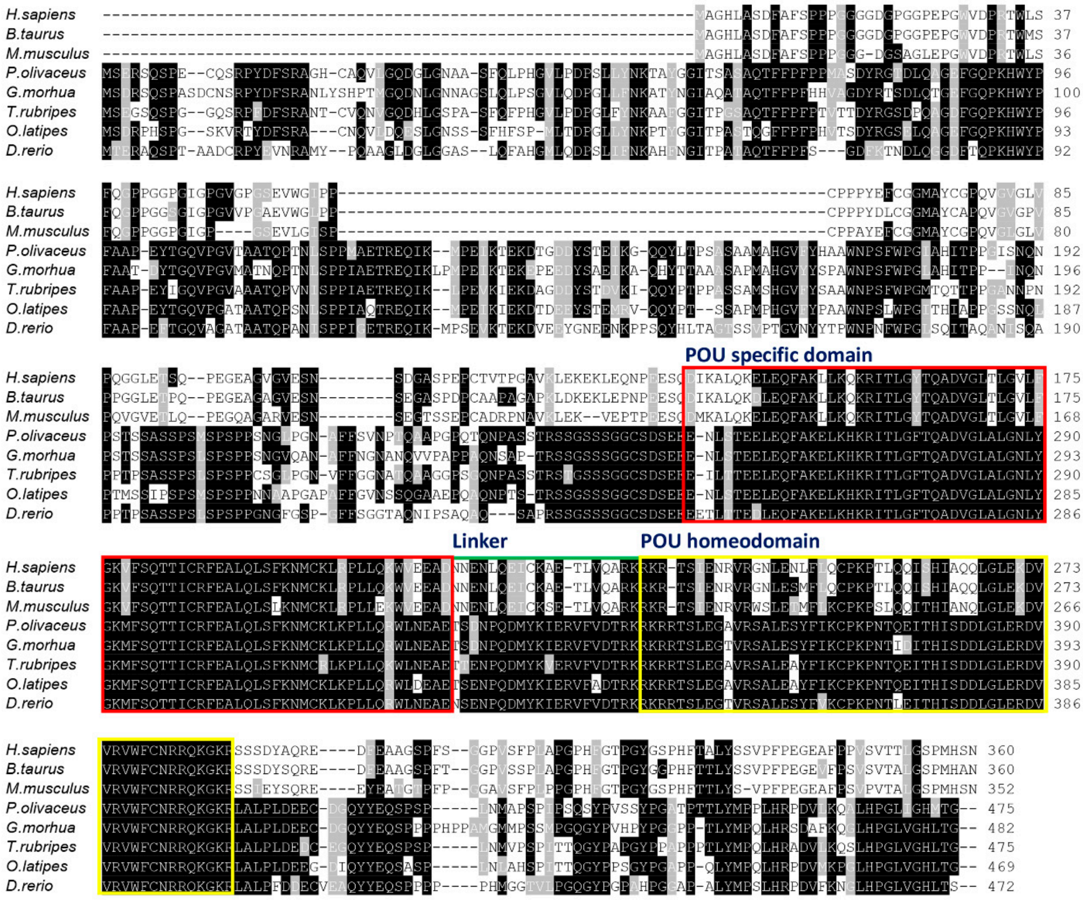

Figure S2. Comparison of the full-length amino acid sequences of Japanese flounder Pou5f3 protein with other vertebrate orthologues. Identical and similar residues are highlighted in black and gray, respectively. POU specific and homeodomains are indicated with red and yellow frames, respectively. The linker regions between two domains are shown by green line. The alignment is generated with ClustalW and shaded with BOXSHADE 3.21. The hyphens (−) indicate gaps that were inserted to maximize sequence similarity.

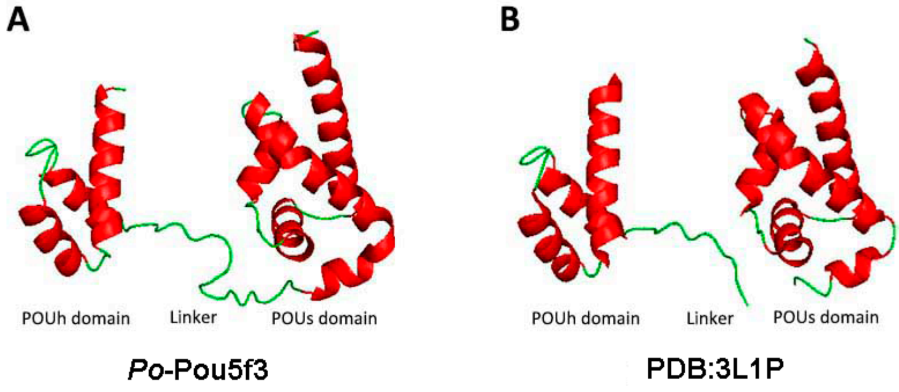

Figure S3. The predicted three-dimensional model of *Po*Pou5f3 POU domain based on *M. musculus* Pou5f1 (PDB ID: 3L1P) by homology modelling method using SWISS-MODEL online server. The α-helices and loop regions are shown as red and green ribbons, respectively.
